# Measuring and controlling medical record abstraction (MRA) error rates in an observational study

**DOI:** 10.1186/s12874-022-01705-7

**Published:** 2022-08-15

**Authors:** Maryam Y. Garza, Tremaine Williams, Sahiti Myneni, Susan H. Fenton, Songthip Ounpraseuth, Zhuopei Hu, Jeannette Lee, Jessica Snowden, Meredith N. Zozus, Anita C. Walden, Alan E. Simon, Barbara McClaskey, Sarah G. Sanders, Sandra S. Beauman, Sara R. Ford, Lacy Malloch, Amy Wilson, Lori A. Devlin, Leslie W. Young

**Affiliations:** 1 Department of Biomedical Informatics, University of Arkansas for Medical Sciences, 4301 W Markham St., #782, Little Rock, AR 72205 USA; 2 School of Biomedical Informatics, University of Texas Health Science Center at Houston, Houston, TX USA; 3 Department of Biostatistics, University of Arkansas for Medical Sciences, Little Rock, AR USA; 4 Department of Pediatrics, University of Arkansas for Medical Sciences, Little Rock, AR USA; 5 University of Texas Health Science Center at San Antonio, Joe R. & Teresa Lozano Long School of Medicine, San Antonio, TX USA; 6 Department of Medical Informatics and Clinical Epidemiology, Oregon Health and Science University, Portland, OR USA; 7 Environmental Influences On Child Health Outcomes (ECHO) Program, National Institutes of Health, Rockville, MD USA; 8 Pittsburg State University, Pittsburg, KS USA; 9 Department of Pediatrics, University of New Mexico Health Sciences Center, Albuquerque, NM USA; 10 Department of Pediatrics, Warren Alpert Medical School of Brown University, Providence, Rhode Island, USA; 11 University of Mississippi Medical Center, Jackson, MS USA; 12 Alaska Native Tribal Health Consortium, Anchorage, Alaska USA; 13 Department of Pediatrics, University of Louisville, Louisville, KY USA; 14 Department of Pediatrics, The Larner College of Medicine at the University of Vermont, Burlington, VT USA

**Keywords:** Medical record abstraction, Data quality, Clinical research, Clinical data management, Data collection

## Abstract

**Background:**

Studies have shown that data collection by medical record abstraction (MRA) is a significant source of error in clinical research studies relying on secondary use data. Yet, the quality of data collected using MRA is seldom assessed. We employed a novel, theory-based framework for data quality assurance and quality control of MRA. The objective of this work is to determine the potential impact of formalized MRA training and continuous quality control (QC) processes on data quality over time.

**Methods:**

We conducted a retrospective analysis of QC data collected during a cross-sectional medical record review of mother-infant dyads with Neonatal Opioid Withdrawal Syndrome. A confidence interval approach was used to calculate crude (Wald’s method) and adjusted (generalized estimating equation) error rates over time. We calculated error rates using the number of errors divided by total fields (“all-field” error rate) and populated fields (“populated-field” error rate) as the denominators, to provide both an optimistic and a conservative measurement, respectively.

**Results:**

On average, the ACT NOW CE Study maintained an error rate between 1% (optimistic) and 3% (conservative). Additionally, we observed a decrease of 0.51 percentage points with each additional QC Event conducted.

**Conclusions:**

Formalized MRA training and continuous QC resulted in lower error rates than have been found in previous literature and a decrease in error rates over time. This study newly demonstrates the importance of continuous process controls for MRA within the context of a multi-site clinical research study.

## Background

Medical record abstraction (MRA) has traditionally been, and continues to be, one of the most common forms of data acquisition for clinical research studies [1]. However, the quality of MRA has often been questioned [2, 3], as it is highly prone to human error [2–6] and often adds to the overall complexity of clinical research [7–12]. For example, studies have shown that error rates associated with MRA are an order of magnitude greater than other data collection methods [2, 3], and the most significant source of error in clinical research [13, 14]. Moreover, the inherent complexities of electronic health records (EHR) often add to the variability of the data abstracted, further contributing to the high and highly variable discrepancy rates associated with MRA [3, 4, 15]. Traditionally, data quality assessments in clinical studies have been limited to database error rates and often overlook errors arising from MRA and from transcription from medical records to the research database [4]. This is unfortunate because MRA remains the dominant method of data collection in retrospective and prospective research.

Although position papers and reports of empirical results exist, the reasons for high error rates associated with MRA have not been systematically studied, and the mechanisms are not clearly understood. Importantly, MRA errors are less likely to be detected by downstream data processing, such as data entry or programmatic data cleaning. For example, an incorrect but plausible value chosen from the medical record will not be detected by valid range checks. MRA errors that result in plausible values will only be detected through comparison to the medical record (i.e., re-abstraction). Accordingly, it is critical that the quality of the data collected through MRA be closely monitored and managed. However, attempts to improve the quality of MRA have not been formally evaluated in the literature. Thus, improvements in MRA data quality have been limited. Our research aims to address this gap by implementing [15] and evaluating a standardized process for MRA training and continuous quality control (QC) within the context of a clinical research study.

As a retrospective chart review, the Advancing Clinical Trials for Infants with Neonatal Opioid Withdrawal Syndrome Current Experience (ACT NOW CE) Study [16] was an example of a clinical study dependent on MRA for data acquisition and subject to data accuracy and quality concerns. Thus, special consideration was given to quality assurance and control of the MRA process for the ACT NOW CE Study [15]. In an attempt to reduce data quality issues, study-specific MRA training was provided to sites prior to activation [15], and a formalized QC process was conducted throughout the course of the study across all participating sites. Throughout the study, data was collected from all participating sites indicating (1) the number of discrepancies per MRA training case, per abstractor, per site, and (2) the number of discrepancies versus true errors per QC, per abstractor, per site.

The objective of this study was to determine the potential impact of formalized MRA training and continuous QC processes on data quality over time and provide a baseline measure for traditional MRA error rates. We hypothesized that the implementation of formalized MRA training and continuous QC monitoring conducted throughout the course of the ACT NOW CE Study would result in (1) improvement in error rates for the ACT NOW CE Study when compared to the acceptable error rate threshold calculated for this study, and (2) improvements in error rates for the ACT NOW CE Study over time. To our knowledge, this is the first time formalized MRA training and continuous QC were implemented and evaluated during the context of a clinical research study.

## Methods

The ACT NOW CE Study was a multi-site, retrospective chart review capturing data from infants born between July 1, 2016 and June 30, 2017 “to inform the design of a clinical trial to improve care and outcomes for infants with neonatal opioid withdrawal syndrome (NOWS)” [16]. Thirty sites from the Environmental influences on Child Health Outcomes IDeA States Pediatric Clinical Trials Network (ECHO ISPCTN) and the *Eunice Kennedy Shriver* National Institute of Child Health and Human Development’s (NICHD) Neonatal Research Network (NRN) distributed across the U.S. participated in the study. The medical records of approximately 1,800 infants with NOWS were abstracted across all sites, of which a subset of cases (over 200) underwent a formalized QC process to identify data quality errors and determine the association between MRA and data quality.

**Fig. 1:**
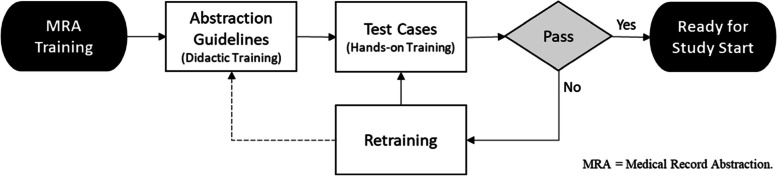
Medical Record Abstraction (MRA) Training Process Flow Diagram

To evaluate the MRA process, continuous QC monitoring was performed throughout the course of the ACT NOW CE Study. This process required a certain percentage of cases at each site to be re-abstracted by a second, independent abstractor from that site. Prior to the start of the ACT NOW CE Study, (1) the acceptable error rate threshold was set (no greater than 4.93% or less than 500 errors per 10,000 fields) [15], (2) a formal abstraction guideline for the study was developed to ensure consistency in data collection across abstractors and sites, and (3) each abstractor (primary abstractor and QC-abstractor) received extensive MRA and QC training (Fig. 1). Additional information on the MRA and QC training process can be found in our prior manuscript [15].

At a minimum, each site performed QC on the first 3 cases abstracted by the site (QC1). Depending on the total number of cases abstracted by the site, additional QC “events” would be required after every 25 cases, one randomly selected case for every 25 cases abstracted and entered into the electronic data capture (EDC) system (QC25, QC50, etc.). Accordingly, the total number of QC Events conducted per site corresponded to the total number of cases abstracted at the site. For example, a site with 25 total cases would be required to QC at least 4 cases (3 for QC1, and 1 for QC25). A site with 125 total cases would be required to QC at least 8 cases (3 for QC1, and 1 each for QCs 25, 50, 75, 100, and 125). Seven distinct QC Events were observed over the course of the study (QC1, QC25, QC50, QC75, QC100, QC125, QC150).

**Fig. 2:**
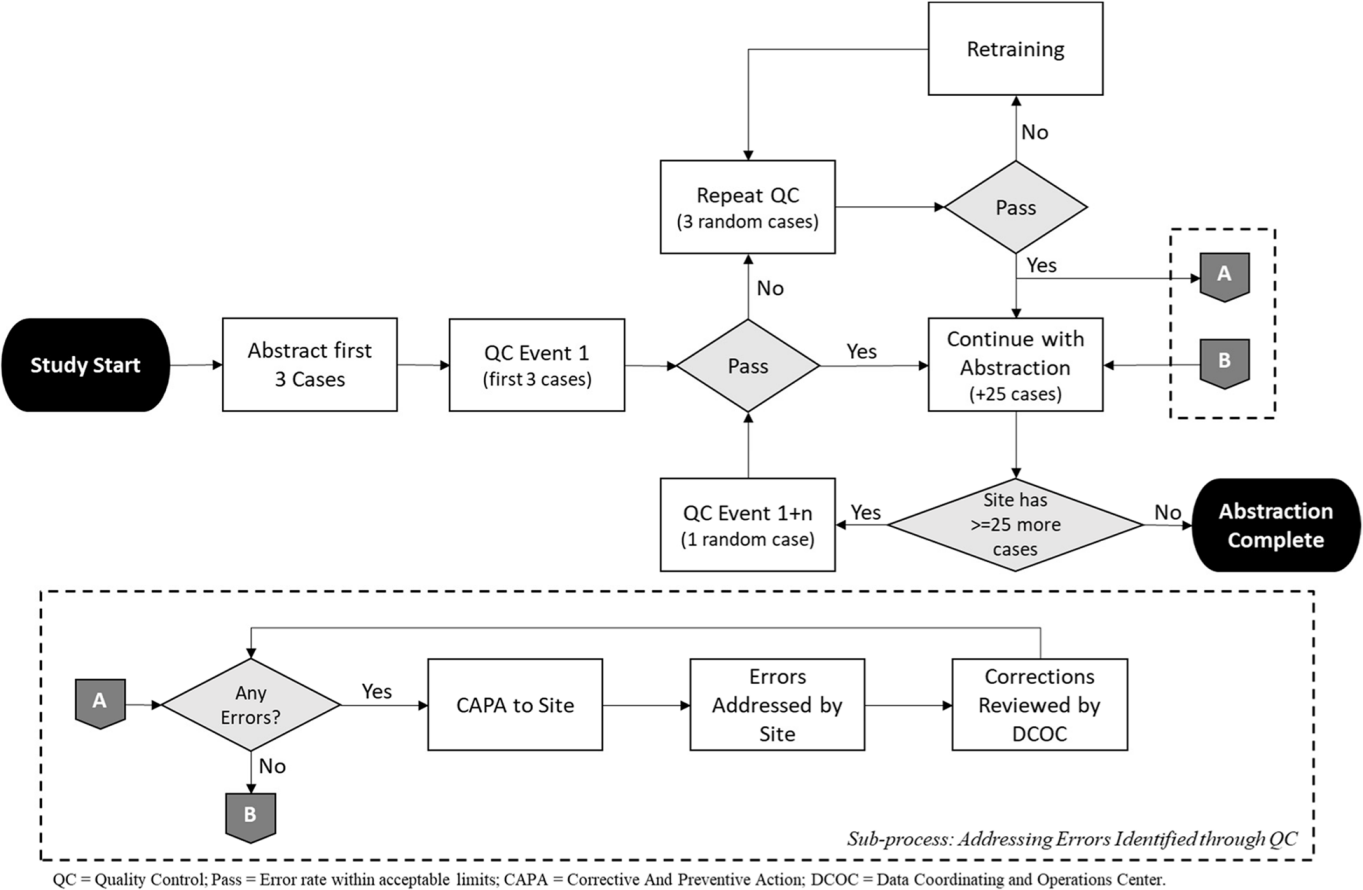
Continuous Quality Control (QC) Process Flow Diagram

A high-level overview of the QC process is described here and depicted in Fig. 2. The primary abstractor would perform MRA on a set of cases (up to 3 for QC1 and an additional 1 case for every QC Event thereafter). The site would notify the Data Coordinating and Operations Center (DCOC) once the specified number of cases had been entered into the EDC and cease all data collection and entry until QC was completed. Using a random number generator, the DCOC identified case(s) for QC and notified the site’s QC-abstractor, who would independently abstract the assigned case(s). The QC-abstractor was not able to see how the primary abstractor identified the data elements within the EHR. Essentially, the QC-abstractor carried out their abstraction and data entry as if it was a completely new case. Upon completion, an automated script was triggered to run, which compared the data entered for each QC case (primary- vs. QC-abstractor) and generated a report with a list of discrepancies.

The system considered any inconsistency between the primary- and the QC-abstractor as a *discrepancy*. By design, the system was highly sensitive to detect any inconsistency in data entry. Once the report was generated, an informaticist and site manager from the DCOC met with the site (both the primary- and QC-abstractors) via video conference, and reviewed the results of the discrepancy report. During the review, sites referenced their EHRs to identify the true value for all discrepancies. The team reviewed each discrepancy and identified true errors. A discrepancy was considered a *true error* if the primary abstractor had entered data into the EDC that was inconsistent with what was in the EHR (the gold standard). If the primary-abstractor’s data entry matched the EHR, the discrepancy was not considered a true error (even if the QC-abstractor did not match). The error rate was then calculated and shared with the site along with a corrective and preventive action plan (CAPA).

**Fig. 3:**
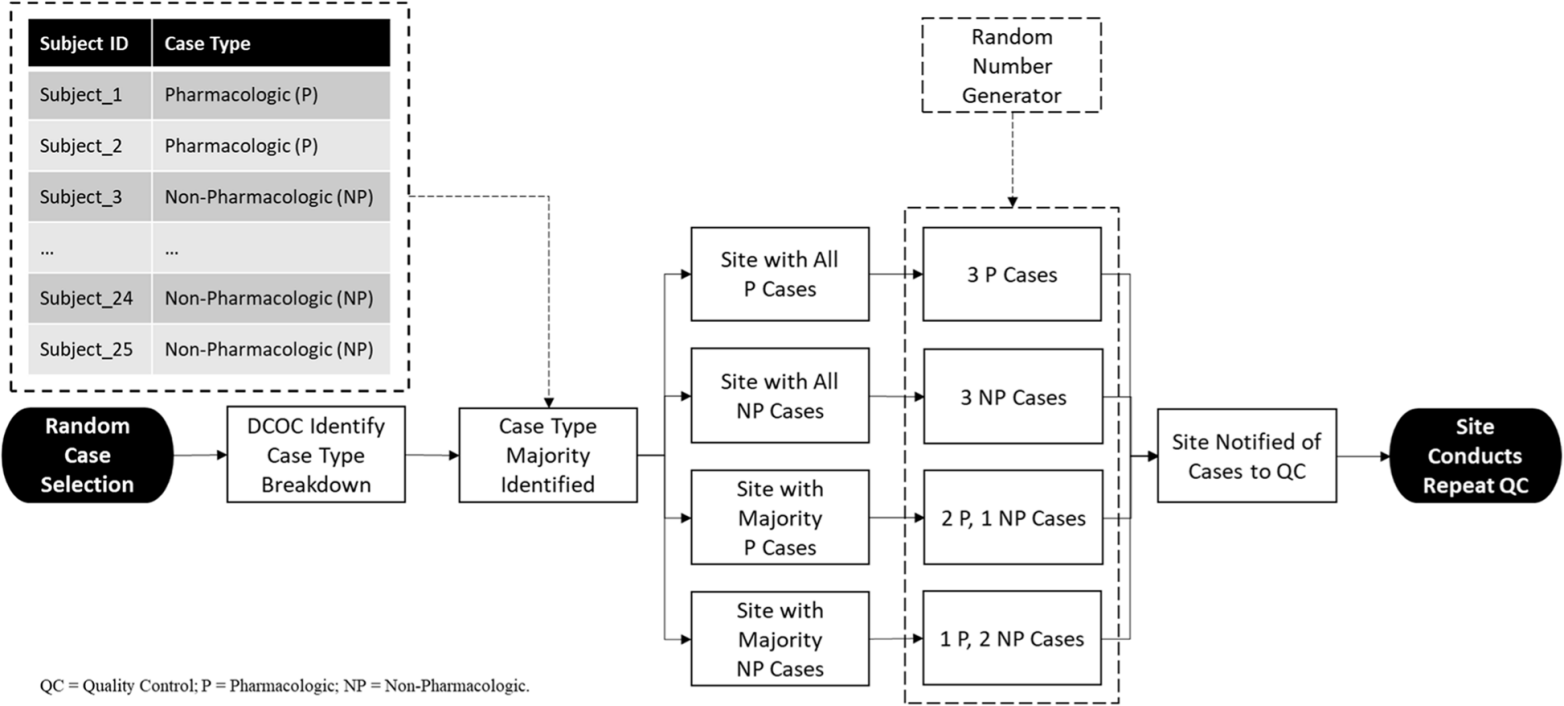
Random Case Selection Process for Repeat Quality Control (QC) Events

In the event that a site exceeded the acceptance criteria for the specified QC Event, the site would be required to repeat the event on another 3, randomly selected cases (essentially increasing the total number of cases undergoing QC at the site). Cases for repeat QC Events were selected by the DCOC as follows (Fig. 3). First, the DCOC identified the treatment type (pharmacologic vs. non-pharmacologic), referred to as the *case type*, most prominent in the current set of abstracted cases at the site, and, then, randomly selected 2 cases of the most prominent case type and 1 case from the other case type for re-abstraction. In situations where the site exceeded the acceptance criteria for the repeat QC, the site (primary- and QC-abstractors) would be required to participate in retraining and perform (and pass) another QC before continuing with the study. If the site was within the acceptable limits, they were able to continue with data collection.

The Error Rate Calculation framework, outlined in the Good Clinical Data Management Practices (GCDMP) guidelines [17], was used to describe error rates, the distribution of the error rates, and the error rates over time. Simply put, error rate is a ratio between the number of data errors detected compared to the total number of data fields collected:

1 $$Error\ Rate=\frac{Number\ of\ Errors\ Detected}{Number\ of\ Fields\ Collected}$$

For this study, we initially calculated the crude MRA error rates along with the Wald’s 95% confidence intervals (CIs) over time. Error rates were calculated using all/total fields (“all-field” error rate) and using only populated fields (“populated-field” error rate) to provide both an optimistic and a conservative measurement, respectively, to account for the variability in the calculation and reporting of error rates in the literature [2–6]. We derived an adjusted MRA error rate along with a 95% CI using a generalized estimating equation model to account for the clustering.

The total number of QC cases was a function of the design and total number of NOWS cases identified by the parent study [16]. Of the 1,808 total NOWS cases, 219 cases were selected for QC based on the methods described above. Four of those cases did not have QC performed due to data entry issues that did not allow for a full QC report to be generated. These were excluded from the analysis. Thus, the analytic sample consisted of 215 QC cases. When calculating the error rates for both the all-field and populated-field totals, the study population was divided into two groups, or *case types*, based on the methods used to treat the infant for NOWS: using pharmacologic therapy (P) or using only non-pharmacologic therapies (NP). The number and types of data elements varied between the two groups (the pharmacologic treatment group requiring more variables), differences that could cause variation in the number of errors. After reviewing the adjusted error rates by case type, the decision was made to combine cases when calculating the changes in error rates over time, as the adjusted error rates by case type did not offer statistically significant results to warrant further investigation. We derived both the crude and adjusted populated-field error rates at each of the 7 distinct QC Event times. For each set of crude and adjusted populated-field error estimates, we fitted separate time series regression with a time trend as the independent variable. We report both the slope estimates and corresponding 95% confidence limits.

## Results

The ACT NOW CE Study electronic case report form was comprised of 312 total data elements. Of the full set (*N* = 312), nearly three-quarters (*n* = 211 [68%]) of the data elements fell within the medication (*n* = 152 [49%]) and medical history (*n* = 59 [19%]) domains. The remaining data elements were distributed relatively evenly across five domains: demographics (*n* = 20 [6%]), diagnosis (*n* = 18 [6%]), eligibility (*n* = 15 [5%]), encounter (*n* = 31 [10%]), and procedure (*n* = 17 [5%]). Table 1 provides a breakdown of total subjects, fields per case, total fields, and populated fields across 2 subgroups (pharmacologic cases and non-pharmacologic cases). Of note, the medication domain captured both concomitant and general medication data on the mother, as well as pharmacologic treatment data on the infant. Thus, some medication-related fields (i.e., concomitant and general medications) were required for both pharmacologic and non-pharmacologic cases (e.g., “Indicate any medication prescribed to the mother during pregnancy for the treatment of opioid dependency.”), and contributed to the field per case totals for both case types noted in Table 1. Across all 215 QC cases, the all-fields count was 48,880 total fields. The populated-field count was 18,843 total fields, a little under half the all-fields count.

**Table 1:** QC Dataset: population breakdown

	Total Subjects n (%)	Fields per Case n	Total Fields n	Populated Fields n
P	85 (40%)	312	26,520	10,425
NP	130 (60%)	172	22,360	8,418
Study Totals	215 (100%)	-	48,880	18,843

*P* Pharmacologic cases, *NP* Non-pharmacologic cases. “Total Fields” was calculated by multiplying the total subjects (column 1) by the number of fields per case (column 2); and was used as the denominator to calculate all-field error rates. “Populated Fields” was calculated by multiplying the total subjects per case type by the total number of fields populated for each subject that fell within that category

Table 2 provides a breakdown of the error rates by case type (pharmacologic and non-pharmacologic) for both the crude and adjusted estimates. When considered in aggregate (*n* = 215 QC cases), a total of 2,394 discrepancies were identified across all cases. Of the 2,394 discrepancies, 573 true errors were identified. Accordingly, the all-field error rate was 1.24%, 95% CI [1.14, 1.34], and the populated-field error rate was 3.04%, 95% CI [2.81, 3.30], across the full QC dataset (across all case types, across all sites). This translated to 124 and 304 true errors per 10,000 fields, respectively. Accounting for clustering, the study total all-field adjusted error rate was 1.17%, 95% CI [0.91, 1.50], and the adjusted populated-field error rate was 2.87%, 95% CI [2.21, 3.74]. The 95% CIs for adjusted error rates were much wider compared to the crude estimates.

**Table 2:** QC Dataset: error rates

	True Errors n	All-Field Error Rate % [95% CI]	Adjusted All-Field Error Rate % [95% CI]	Populated-Field Error Rate % [95% CI]	Adjusted Populated-Field Error Rate % [95% CI]
P	273	1.06 [0.94, 1.20]	1.07 [0.81, 1.42]	2.62 [2.33, 2.94]	2.64 [1.97, 3.54]
NP	300	1.45 [1.30, 1.63]	1.35 [1.04, 1.75]	3.56 [3.19, 3.98]	3.31 [2.53, 4.33]
Study Totals	573	1.24 [1.14, 1.34]	1.17 [0.91, 1.50]	3.04 [2.81, 3.30]	2.87 [2.21, 3.74]

All-Field Error Rate was calculated using the Total Fields count, and Populated-Field Error Rate was calculated using the Populated Fields count from Table 1

Table 2 also presents error rate estimates stratified by case type for both all-field and populated field. Using the crude estimates, the differences between the error rates for non-pharmacologic versus pharmacologic cases based on all-field and populated-field were statistically significant (${\Delta }_{all-field}=0.39;p=0.0002$ and ${\Delta }_{populated}=0.95;p=0.0002)$. In contrast, after accounting for the clustering, the differences in adjusted error rates among the all-field and populated-field were not statistically significant (significant (${\Delta }_{all-field}=0.28;p=0.152$ and ${\Delta }_{populated}=0.67;p=0.269)$.

**Fig. 4:**
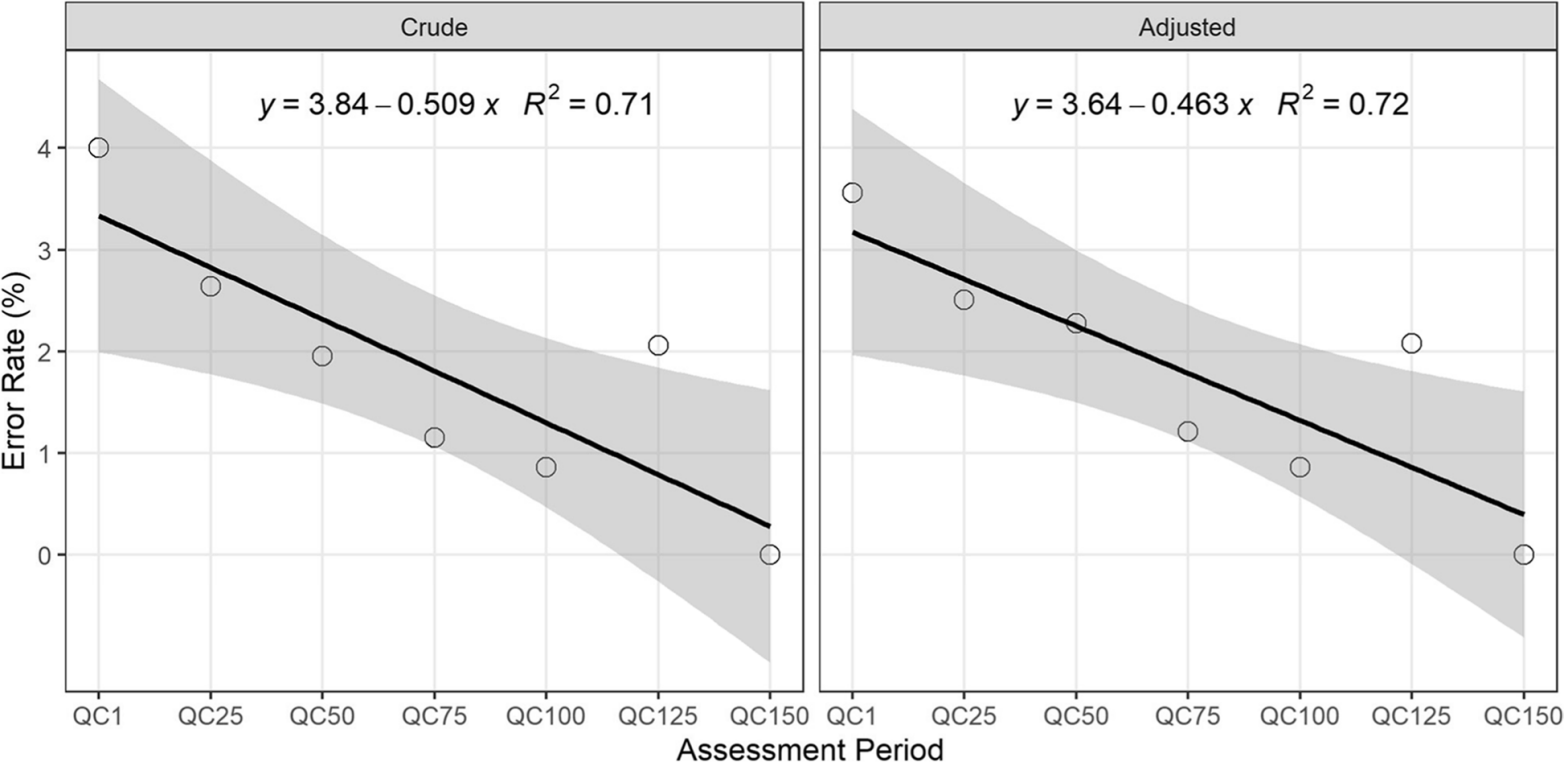
Error Rates Over Time for the ACT NOW CE Study. Note. Regression analysis performed on the “crude” error rates was based on Eq. (1) using only populated fields. Regression analysis performed on the “adjusted” error rates was based on error rates derived from a generalized estimating equation model to account for clustering

The error rates for the ACT NOW CE Study over time are displayed in Fig. 4. For both the crude and adjusted populated-field error rates, there was a statistically significant downward trend among the sites with multiple QC Events. More specifically using the crude error estimates, the error rate decreased by 0.51 percentage points (*p* = 0.017; 95% CI: [-0.88%, -0.14%]; *R*^*2*^ = 0.71) for each additional QC Event. Similarly, the error rates accounting for clustering decreased by 0.46 percentage points (*p* = 0.016; 95% CI: [-0.80%, -0.13%]; *R*^*2*^ = 0.72) for each additional QC Event.

## Discussion

In this analysis, we found that error rates using a formalized MRA training and continuous QC process were on the order of 1–3% (or 100 to 300 errors per 10,000 fields), depending on whether the optimistic, all-field or the conservative, populated-field approach was used to determine error rates. Using either approach, these error rates were substantially lower than the pre-determined acceptable error rate limit set for the ACT NOW CE Study. The use of standardized MRA training and QC, consistently deployed by the ACT NOW CE Study, allowed for greater control in the variability of error rates across sites and over time. Further, a clear pattern of decline in error rates was observed over time (on average, –0.51 percentage points) as participating sites continued to perform QC throughout the course of the study. Based on these results, it appears that using formalized MRA training and continuous QC processes has the potential to positively affect the data quality in a clinical research study by maintaining lower error rates overall and reducing error rates over time, ultimately, reducing how often the quality of MRA is questioned [2, 3].

We speculate that the reduction in error rates could be partially attributed to the continuous learning that occurs with each QC Event, as each event provided another opportunity for study coordinators to address questions pertaining to the abstraction guidelines and/or the data entry process. This gave all sites a chance to reset with each QC Event and ensure that the data abstraction and entry moving forward were in accordance with the standards set forth by the study. Corrective and preventive action (CAPA) plans were also provided after each QC Event to guide sites for how to handle identified errors, data entered previously (if applicable), and data entry moving forward. Plans aligned with best practices, outlined in the GCDMP guidelines [17].

To a degree, the fact that the continuous QC process occurred throughout the course of the study (as opposed to at the end, as in the case with traditional site monitoring), may have also contributed to the lower error rates, the idea being that sites were aware of and expecting the QC to occur and may have been more prepared for abstraction and entry. However, in most cases, most studies do employ some method of QC (e.g., database checks, statistical checks, and traditional site-level monitoring) of which the study team and sites are made aware. In general, the clinical operations, informatics, and/or biostatistics teams work regularly with sites at various points throughout the study to address discrepancies identified via queries and site monitoring visits. Therefore, site personnel are typically aware of the importance of and are targeting quality data entry. Further, this awareness does not preclude sites from experiencing data entry errors due to a misinterpretation of the variables and their location in an EHR, given that the more manual elements of MRA are highly susceptible to human error [2–6]. This is why the abstraction training and formal abstraction guidelines are critical to improving both competency and performance of abstractor(s) in reducing error rates.

We offer several possible explanations for the variability in error rates across clinical research studies and sites, which could inform modifications of clinical research practice. The experience of the abstractor(s) and their level of familiarity with the EHR may affect the resulting error rate. For example, in the case of the ACT NOW CE Study, several abstractors were registered nurses who were familiar with the population and experienced with where the data would exist within their institutional EHRs. Another possible explanation is data complexity. For the ACT NOW CE Study, data complexity varied by case type. Pharmacologic data elements (e.g., medication-dosing information) tended to be discrete fields, often more consistently documented within the EHR. In comparison, non-pharmacologic data elements included items that were much more difficult to find in the EHR, as they could be documented in a variety of places as free-text (e.g., in clinical notes and/or flowsheets). As such, data abstractors often struggled as they searched for the unstructured fields, which is likely the reason the error rates were slightly higher for NP versus P cases. Other factors worth noting include the local system implementations and workflows, the number of data processing steps, and the differences in reporting error rates.

That said, to help mitigate any variability in current clinical research practice, we specifically recommended that sites use their most experienced coordinator as the primary abstractor (those with the most knowledge on NOWS cases and the most familiarity with where the data is located within the EHR), and, if possible, a coordinator with extensive experience handling NOWS data within the EHR as the QC abstractor. The purpose for this was to set the sites up for success early on and an attempt to reduce variability across sites and keep error rates low from the start. This approach is something we recommend for our studies in general, as a best practice, in cases where MRA and traditional site monitoring approaches are used. To address the data complexity challenges of abstractors, current clinical research practice could be modified with the development of training and job aids for abstractors (e.g., developing universal schemas, maps, or training aids that provide guidance on where non-pharmacologic data elements are commonly located within EHR systems). We would recommend that other researchers follow suit when taking on retrospective, MRA-based studies.

Ultimately, the decisions to employ study-specific abstraction training and continuous QC resulted in significantly lower error rates overall, with continued improvements in data quality observed over time. MRA training conducted prior to study start offered sites with a clear set of instructions for identifying the appropriate study data elements within the EHR, while the use of continuous QC during the study provided a mechanism for catching and addressing errors early in the data collection process and provide retraining (as necessary) and corrective action for future abstraction. Importantly, the results presented here are of immediate use in informing investigators and research teams as they plan and execute future clinical research studies. The framework used in the ACT NOW CE Study for controlling MRA data errors can be leveraged by other researchers going forward. Thus, we recommend this work be used to inform future study design and quality assurance processes for clinical studies relying on MRA for data collection.

### Assessing the impact of continuous QC on workload

While we did not formally measure the workload associated with the QC process, we are able to provide a description of the additional resources (personnel) required, as well as general effort estimates based on the number of QC Events and the number of personnel involved in the QC process for the ACT NOW CE Study. Here, we aim to provide a high-level view of the requirements imposed on the coordinating center (responsible for designing and operationalizing the QC process) and study sites (responsible for complying with the QC process) to demonstrate the potential burden undertaken by this study, as well as the possible improvements or reduction of work that could result from such an implementation.

With regards to personnel, we required sites to have at least two coordinators (one for the primary abstraction and one for the QC abstraction). In many cases, this did not affect the personnel count for the sites, as the sites had planned for multiple coordinators to share the abstraction work. Beyond that, no additional personnel were required of the site. From the perspective of the coordinating center, the DCOC had already accounted for three site managers who would each be responsible for managing 10 sites each (30 total sites). On the informatics side, we did rely on a programmer to assist in the development of the technical components to support the added QC functionality, as the DCOC maintained and managed its own EDC and QC system internally. That said, the number of personnel did not change; instead, an additional task was assigned to existing staff (taken into account in the time burden below). For those relying on vended software or products, these development responsibilities would likely fall on the vendor.

With regards to time, for each QC Event, sites were required to (1) repeat abstraction for the case(s) selected for QC, (2) attend a QC meeting with the DCOC staff to review the discrepancies, and (3) spend time correcting any true errors and carrying out any tasks outlined within the CAPA. As the QC caseload at any given time point was minimal (typically 1 case), the burden imposed by the repeat abstraction was likely negligible for the average NOWS case. Pharmacologic cases would likely take longer than non-pharmacologic cases due to the number of required fields (312 vs. 172); although, typically, the extra pharmacologic data points followed a pattern and were often captured in the EHR as discrete, structured data fields (e.g., medication dates and doses), as opposed to non-pharmacologic cases, which tended to have data located in unstructured fields (e.g., text in flowsheets or nurses notes). Therefore, for the typical NOWS case, regardless of case type, we considered the extra effort (time spent on re-abstraction) relatively equal.

Where the timing required for re-abstraction might deviate would be in situations where either (1) the case requiring QC was not a typical case or (2) the site had to perform multiple repeat QC Events. We considered a typical case to be one that was born in-house (not a transfer case) with straightforward data entry (minimal or easy to identify unstructured data and consistency in documentation of discrete/structured data). Any deviation from the typical case – for example, cases requiring primarily unstructured data that is inconsistently documented in the EHR – would increase the amount of time required to re-abstract the case for QC. Additionally, sites requiring repeat QC Events (having exceeded the acceptable error rate threshold during the original QC Event) would also be susceptible to increases in time/effort requirements, as this would increase the re-abstraction by 3 cases with each repeat QC Event. Of the 30 total sites, 12 unique sites required repeat QC Events, averaging between 1–2 repeat QC Events requiring re-abstraction of 3–6 additional cases. The effects across sites would vary depending on their unique caseloads. For example, a site with 10 or fewer cases requiring no repeat QC Events would be responsible for 3 total QC cases. If 2 repeat QC Events were required for this site, 6 additional cases would be required, totaling 9 QC cases of 10 total cases – essentially doubling their workload. In contrast, a site with 100 cases would be impacted less in this situation – originally requiring 7 QC cases, plus 6 additional cases for having to repeat 2 QC Events, totaling 13 QC cases out of 100 (or an increase in effort by 13%).

With regards to the time requirement for QC meetings, each QC Event typically required only a single meeting (60 min) with both study coordinators, the assigned site manager, and an informatics analyst. Again, the effects on effort would vary by site based on the total number of QC Events encountered. Thus, a site with fewer QC Events would spend less time than a site with more QC Events. For example, a site with only a single QC Event (QC1) would only need to allocate an additional hour of effort for QC meetings. In contrast, a site requiring all 7 QC Events (sites with cases greater than or equal to 150) would need to allocate an additional 7 h for QC meetings. The number and/or duration of QC meetings could potentially increase for QC Events having a larger number of total discrepancies for review. This would be something for other researchers to consider when planning out site effort.

Effort for time spent post-QC for error correction would also vary by site, by QC Event. This process is akin to traditional discrepancy management practices used to handle system-generated queries for pre-programmed data validation checks. Thus, the total number of true errors identified (which would be entered into the EDC as queries) would affect the total time required. For example, a site for which a QC Event yielded 3 true errors would require much less time to correct than a site with a QC Event yielding 10, 15, or 20 true errors. Clear instructions within the queries generated in the EDC and articulation in the CAPA plan of site responsibilities and requirements can help ease the burden on the site.

For the DCOC, additional time was required to program the QC functionality into the EDC, to review QC reports in preparation for the QC meeting, to conduct the QC meeting, and to generate the post-QC report (including inputting queries and generating the CAPA plan). Once system requirements were identified, programming time was relatively small, but did encompass coding, testing, rework (as needed), and final implementation. The discrepancy identification and report generation was automatic and system-generated, so no additional time was required for these tasks. Preparation for QC meetings was minimal (not typically more than 30 min per QC Event), and the QC meeting time for the DCOC was equal to that for the site (60 min per QC Event). Depending on the results of the QC Event (the number of true errors and the corrections required), the post-QC report could take between 30–90 min).

Due to the nature of the continuous QC process and the DCOC’s constant monitoring of study data throughout the course of the study, we were able to forgo traditional, data-centric study monitoring processes. While we had a study monitor on staff, we were able to greatly reduce the amount of effort required of the monitor for this particular study. Traditionally, study monitors are tasked with performing in-person or virtual site visits for the purpose of reviewing and evaluating site-level performance and study conduct. Often, this includes a detailed review of the data entered, requiring the monitor to perform source data verification on either the full set or a predetermined subset of the data (which will vary by study and funding agency requirements). This would typically happen once during the course of a study this size. However, the continuous QC process fulfilled these specific monitoring needs (i.e., the data review component), and allowed us to shift the way we operationalized study monitoring – monitoring at multiple time points and addressing data issues as they arise. Further, traditional site requirements were also reduced (sites also play a major role in the study monitoring process), albeit some of this effort was shifted over to allow for continuous QC.

### Limitations

The limitations of this work are as follows. First, it is important to note that the values presented pertaining to the impact on workload and burden are all estimates and could vary by study, by site, and by QC Event. As we did not formally measure or evaluate burden of continuous QC for this study (not in scope), we are unable to provide concrete numbers for time and effort spent by the sites and the coordinating center. That said, these estimates are based our actual experience carrying out these tasks as study coordinators (BM, SGS, SSB, SRF, LM, AW) and informaticists (MYG, ACW) for the ACT NOW CE Study.

Additionally, the results presented are based on a single, pediatric case study, the ACT NOW CE Study. This is mitigated by the fact that there were 30 sites, which allowed us to assess variability in error rates among multiple sites. Still, the generalizability of this study may be limited by nuances specific to the study sites (e.g., prior expertise, team size, EHR functionalities). Therefore, we recommend that researchers utilize this framework and conduct more systematic data quality analyses for their studies. We also encourage researchers to publish these results to contribute to the larger body of data quality literature and provide additional use cases for the clinical research community. Research from multiple epistemological stances would provide valuable information to confirm or challenge the results identified here.

Given these limitations and the study design, we draw conclusions only about associations and trends. Importantly, although there is strong correlation between use of the MRA-QC framework and our ability to maintain relatively low error rates and continued improvements (reduction of error rates) over time, this association does not imply causality, and other important factors not assessable here may be responsible for these results.

### Future directions

As data (increasingly captured electronically) are used to support direct patient care, performance measurement, and research, the effects of data quality on decision-making need thorough exploration, as do the effects of system usability and data entry and cleaning methods on data quality and clinical workflow. Research opportunities exist (1) to understand the types of errors that perpetuate in MRA data collection, and (2) in the areas of data and process standardization [18–20] to aid in streamlining data collection for clinical research studies. Our ongoing work aims to address these issues as we develop solutions to streamline data collection processes and improve data quality in clinical research. We are currently working on evaluating standards-based mechanisms that could semi-automate the data collection process (reducing the burden of manual MRA) and for continuous data quality improvement. Final analysis is underway and a publication is anticipated for mid- to late-2022.

## Conclusion

Through this work, we have determined the effects of formalized MRA training and continuous QC within the context of a multi-site clinical research study and provided a baseline measure for traditional MRA error rates. More importantly, we have demonstrated that use of a standardized training program and ongoing data quality monitoring processes can reduce error rates. For the ACT NOW CE Study, specifically, the results were twofold: (1) error rates were more controlled and well within the acceptable error rate limits calculated for this study, and (2) the average rate of change over time indicates a decrease in the number of true errors observed over time that may be contributed to the QC training. From these results, it is clear that formalized MRA training and continuous QC conducted throughout the course of a clinical study has the potential to significantly lower error rates overall and over time.

## Data Availability

The datasets generated and/or analyzed during the current study are available in the NICHD Data and Specimen Hub (DASH) repository, https://dash.nichd.nih.gov/study/229026.

## Abbreviations

MRA: Medical Record Abstraction
QC: Quality Control
EHR: Electronic Health Record
ACT NOW CE: Advancing Clinical Trials for Infants with Neonatal Opioid Withdrawal Syndrome Current Experience
NOWS: Neonatal Opioid Withdrawal Syndrome
ECHO: Environmental influences on Child Health Outcomes
ISPCTN: IDeA States Pediatric Clinical Trials Network
NICHD: National Institute of Child Health and Human Development
NRN: Neonatal Research Network
EDC: Electronic Data Capture
QC1: First QC Event: required of all sites on the first 3 cases abstracted and entered into the electronic data capture system
QC25: Second QC Event: required of sites with ≥ 25 total cases, once the 25^th^ case has been abstracted and entered into the electronic data capture system
QC50: Third QC Event: required of sites with ≥ 50 total cases, once the 50^th^ case has been abstracted and entered into the electronic data capture system
QC75: Fourth QC Event: required of sites with ≥ 75 total cases, once the 75^th^ case has been abstracted and entered into the electronic data capture system
QC100: Fifth QC Event: required of sites with ≥ 100 total cases, once the 100^th^ case has been abstracted and entered into the electronic data capture system
QC125: Sixth QC Event: required of sites with ≥ 125 total cases, once the 125^th^ case has been abstracted and entered into the electronic data capture system
QC150: Seventh QC Event: required of sites with ≥ 150 total cases, once the 150^th^ case has been abstracted and entered into the electronic data capture system
DCOC: Data Coordinating and Operations Center
GCDMP: Good Clinical Data Management Practices
CI: Confidence Interval
P: Pharmacologic
NP: Non-pharmacologic
CAPA: Corrective And Preventive Action
